# Applications of linking PBPK and PD models to predict the impact of genotypic variability, formulation differences, differences in target binding capacity and target site drug concentrations on drug responses and variability

**DOI:** 10.3389/fphar.2014.00258

**Published:** 2014-11-26

**Authors:** Manoranjenni Chetty, Rachel H. Rose, Khaled Abduljalil, Nikunjkumar Patel, Gaohua Lu, Theresa Cain, Masoud Jamei, Amin Rostami-Hodjegan

**Affiliations:** ^1^Simcyp Limited (a Certara Company), Blades Enterprise CentreSheffield, UK; ^2^Manchester Pharmacy School, University of ManchesterManchester, UK

**Keywords:** PBPK linked PD models, CYP P450 genotypes and response, heart drug concentration and QTc, formulation effects on drug response, target site concentrations and response

## Abstract

This study aimed to demonstrate the added value of integrating prior *in vitro* data and knowledge-rich physiologically based pharmacokinetic (PBPK) models with pharmacodynamics (PDs) models. Four distinct applications that were developed and tested are presented here. PBPK models were developed for metoprolol using different CYP2D6 genotypes based on *in vitro* data. Application of the models for prediction of phenotypic differences in the pharmacokinetics (PKs) and PD compared favorably with clinical data, demonstrating that these differences can be predicted prior to the availability of such data from clinical trials. In the second case, PK and PD data for an immediate release formulation of nifedipine together with *in vitro* dissolution data for a controlled release (CR) formulation were used to predict the PK and PD of the CR. This approach can be useful to pharmaceutical scientists during formulation development. The operational model of agonism was used in the third application to describe the hypnotic effects of triazolam, and this was successfully extrapolated to zolpidem by changing only the drug related parameters from *in vitro* experiments. This PBPK modeling approach can be useful to developmental scientists who which to compare several drug candidates in the same therapeutic class. Finally, differences in QTc prolongation due to quinidine in Caucasian and Korean females were successfully predicted by the model using free heart concentrations as an input to the PD models. This PBPK linked PD model was used to demonstrate a higher sensitivity to free heart concentrations of quinidine in Caucasian females, thereby providing a mechanistic understanding of a clinical observation. In general, permutations of certain conditions which potentially change PK and hence PD may not be amenable to the conduct of clinical studies but linking PBPK with PD provides an alternative method of investigating the potential impact of PK changes on PD.

## INTRODUCTION

Physiologically based pharmacokinetic (PBPK) modeling provides a mechanistic platform for the integration of the concentration-time profile of the drug with realistic physiological and biological processes in the body. This modeling approach offers an advantage over traditional compartmental modeling approaches since it potentially allows for extrapolation and further investigation into conditions for which pharmacokinetic (PK) studies have not been conducted, thereby informing and accelerating the drug development process. Predictions on drug–drug interactions, first in man dosing, optimal clinical study designs, dosage requirements for drugs that are metabolized by polymorphic enzymes and dosage adjustments in disease states are some of the PBPK applications that could potentially be used during the drug development and regulatory submission processes (Chen et al., 2012; Huang and Rowland, 2012; Rostami-Hodjegan, 2012; Sinha et al., 2012; Zhao et al., 2012; Rowland, 2013; Vieira et al., 2014).

Since the primary concern in drug development is the efficacy and safety of the drug, PBPK linked pharmacodynamic (PD) models can be very valuable, offering a platform for exploring the effect of variability in various physiological, biochemical, and formulation factors on the response to the drug, especially where clinical studies have not or cannot be conducted. An important advantage of PBPK linked PD models is the ability to link the drug concentration at the probable site of action with toxicological and/or therapeutic effect. This is especially important when the plasma concentration is not a good surrogate for the concentration at the site of drug action (Rostami-Hodjegan, 2013), as demonstrated in a recent study with rosuvastatin, a cholesterol lowering drug which is a substrate of the OATP1B1 influx transporter (Rose et al., 2014). A PBPK/PD model that used liver concentrations of rosuvastatin demonstrated improved ability to capture the effect of the OATP1B1 c.521T > C single nucleotide polymorphism on the change in cholesterol synthesis rate in response to rosuvastatin, compared to a model using plasma concentrations of the drug. Based on significant plasma concentration differences, a dosage adjustment in rosuvastatin may have been considered in patients with the OATP1B1 c.521T > C polymorphism, but a clinical study had shown no significant differences in response in the two OATP1B1 phenotypes. However, using PBPK modeling with a PD model driven by free liver concentrations of rosuvastatin, no net difference in liver concentrations of the drug in patients with the polymorphism was observed and their clinical response was similar, suggesting that dosage adjustments were unnecessary.

Many other scenarios in drug development and clinical practice can benefit from incorporating the prior systems knowledge into PBPK models when these are linked to PD modeling. Four such examples from distinct areas are presented in this paper to demonstrate the wide range of applications of this approach. The first case study considers CYP genotypes which can have a significant effect on drug PK. This case study is a quantitative prediction of the variation in the clinical response (measured as heart rate) to standard doses of metoprolol in ultrarapid metabolizers (UMs), extensive metabolizers (EMs), and poor metabolizers (PMs) of CYP2D6. The PBPK model used differences in CYP2D6 abundance obtained from *in vitro* studies to simulate phenotypic differences in metoprolol PK. The second case study explores the potential for the application of PBPK/PD modeling to predict the response to a controlled release (CR) formulation using *in vitro* data for the CR formulation. A PBPK/PD model was developed and verified for a nifedipine immediate release (IR) formulation. It was then used for prediction of the PK and PD profiles of the CR formulation using only the dissolution profile of the CR formulation. The third case study investigates the application of a PBPK model linked to a semi-mechanistic PD model developed for one drug to predict the response to a second drug based on clinical data for the first drug that acts on the same target. A PBPK/PD model was developed and verified for triazolam using an operational agonism PD model. A zolpidem specific target binding parameter obtained by *in vitro* studies was then used with the triazolam model to predict response to zolpidem. Such models are useful for comparing several potential drug compounds that belong to the same therapeutic class, when clinical data is available for just one of the compounds. In the fourth case study, the PD model is driven by target site drug concentrations and used to gain a mechanistic understanding of a clinical observation. The differences in the potential for QT prolongation by drugs such as quinidine in Caucasian and Asian females are well known, although the reason for this difference has not been established. Using PBPK models with heart concentrations of quinidine as the input to PD models, a higher sensitivity to heart concentrations of quinidine was demonstrated in Caucasian females.

## MATERIALS AND METHODS

The Simcyp population-based simulator (V12 http://www.simcyp.com; Jamei et al., 2009) was used in the development of the PBPK/PD models and the simulations. Simcyp compound files (parameters listed in **Tables 1–4**) that were validated previously (Howgate et al., 2004; McGinnity et al., 2008; Polasek et al., 2010; Rowland Yeo et al., 2004; Patel et al., 2014) and population data (Jamei et al., 2014) available in Simcyp (V12) were used. Clinical data used in the case studies were digitized from published clinical studies using the Getdata software. Simulations using the developed models were verified by comparison with clinical data prior to further predictive applications. Simulations were found to be acceptable if the predicted parameters were within two fold of the observed data (Guest et al., 2011) and visual predictive checks showed observed data within the 5 and 95% percentiles of the predicted data. Methodological details relevant to the individual case studies are described below.

**Table 1:** Parameters used for the Metoprolol PBPK model (Simcyp V12).

Parameter	Value	Unit
Molecular weight	267.4	g/mol
LogP	1.88	
Compound type	Monoprotic base	
pKa	9.75	
Blood:plasma	1.15	
Fu plasma	0.88	
Main binding protein	albumin	
Absorption	First order absorption	
fa	1	
ka	1.43	
Distribution	Minimal PBPK model	
Vss	3	L/kg
**Elimination**		
CYP2D6:o-demethylation CLint	39.33	pmol/min/mg microsomal protein
CYP2D6:alpha-OH CLint	8.7	pmol/min/mg microsomal protein
CYP3A4:o-demethylation CLint	3.18	pmol/min/mg microsomal protein
CYP3A4:alpha-OH CLint	0.39	pmol/min/mg microsomal protein
CL_R_	5.23	L/h

**PD model**	Simple Emax model	

Emax	–43.2	Beats/min
EC50	0.13	μM
Baseline	143	Beats/min

### CASE STUDY 1: ASSESSING THE IMPACT OF GENOTYPICALLY CONTROLLED ELIMINATION

Although plasma concentrations of metoprolol and effects on heart rate have been shown to correlate significantly with CYP2D6 metabolic phenotype in clinical studies (Kirchheiner et al., 2004; Sharma et al., 2005), the prevalence of some phenotypes may not be adequately high in a study population to discern the differences in PK and PD. Therefore, it would be of value to use the prior *in vitro* information on metabolism together with PK and PD information in prevalent phenotypes of CYP2D6 to conduct virtual clinical studies with a view to assess the potential pharmacological differences in various less frequent phenotypes, prior to the conduct of clinical studies or in lieu of such studies when the studies are not feasible and yet providing a recommendation is more prudent than leaving a void in prescribing information.

The reduction in heart rate due to a standard 100 mg dose of metoprolol in virtual healthy Caucasian populations was simulated and stratified for their CYP2D6 phenotypes. The Simcyp metoprolol compound file (**Table 1**) was used with a minimal PBPK model, first order absorption and elimination by enzyme kinetics. The study design was matched to that of Kirchheiner et al. (2004). Simulated contribution of the CYP2D6 phenotypes (EM, PM, and UM) to metoprolol PK within Simcyp is based on the propagation of the differences in CYP2D6 abundance, obtained from *in vitro* data. Concentration-time profiles published by Kirchheiner et al. (2004) and Sharma et al. (2005) were compared with the predicted profiles and PK parameters. PD differences in the different phenotypes were assessed by an Emax model published by Kirchheiner et al. (2004) and assumed to be the same regardless of CYP2D6 genotype. Based on this direct effects PD model, response was propagated via changes in the plasma concentration profile. PD simulations were compared with clinical observations from the above two studies, to verify that the model predicted the PD corresponding to the different phenotypes adequately.

### CASE STUDY 2: ASSESSING THE CONSEQUENCES OF MODIFYING THE DRUG FORMULATION

Nifedipine is a dihydropyridine calcium channel blocker commonly used in the treatment of hypertension and exerts its hypotensive effect primarily through arterial dilation. CR formulations are now recommended in the treatment of hypertension as they have been shown to offer a number of clinical benefits over IR nifedipine (Reitberg et al., 1987; Schug et al., 2002; Meredith and Elliott, 2004; Wonnemann et al., 2006). This study aimed to integrate the PBPK models describing the plasma profile of IR and that for the CR nifedipine (Shimada et al., 1996; Brown and Toal, 2008) with the PD model available for IR nifedipine (Shimada et al., 1996), to identify whether it could be extrapolated to predict the response to nifedipine GITS, a CR formulation that is reported to achieve a zero order release rate sustained over 24 h, through an osmotic release mechanism (Brown and Toal, 2008).

The PK and PD profiles for nifedipine in the treatment of hypertension were simulated using the Simcyp nifedipine compound file (**Table 2**), a minimal PBPK distribution model and elimination by enzyme kinetics. To simulate the PK profile of IR nifedipine the first order absorption model was used, while for the nifedipine GITS [a CR formulation reported to achieve a zero order release rate sustained over 24 h through an osmotic release mechanism (Brown and Toal, 2008)] formulation effects were described by a mechanistic absorption model within Simcyp (ADAM) using *in vitro* data (dissolution data) for the CR profile and intrinsic solubility of nifedipine (Janssen Therapeutics, 2013). The PD model relating the nifedipine plasma concentration to the change in systolic blood pressure was a dynamic binding PKPD model, as described by Shimada et al. (1996). Parameters used in the PD model are shown in **Table 2**. Simulated study design was matched to that reported for clinical studies, including age, proportion of females and fasted or fed state dosing. Ethnicity was also matched to the clinical study using the built in Simcyp Japanese and North European Caucasian populations. Where ethnicity of study subjects was not reported it was assumed based on the location of the approved study site or the country of residence of the study authors. Using the developed PBPK/PD models, concentration and response profiles were simulated for two different doses of the GITS formulation and compared with clinical data.

**Table 2:** Parameters used for the Nifedipine PBPK model (Simcyp V12).

Parameter	Value	Unit
Molecular weight	346.3	g/mol
LogP	2.69	
Compound type	Monoprotic base	
pKa	2.82	
Blood:plasma	0.685	
Fu plasma	0.039	
Main binding protein	albumin	
Absorption	First order absorption for IR nifedipine Mechanistic absorption model (ADAM) for the CR formulation	
fa	1	
ka	3.67	1/h
Distribution	Minimal PBPK model	
Vss	0.57	L/kg
**Elimination**		
CYP3A4:oxidation Km	10.5	μM
CYP3A4:oxidation Vmax	22	pmol/min/mg microsomal protein
CYP3A5:oxidation Km	31.9	μM
CYP3A5:oxidation Vmax	3.5	pmol/min/mg microsomal protein
CL_R_ (renal clearance)	0	L/h

**PD model**	Dynamic binding model with empirical transduction	

k_on_ (rate constant for binding of drug to receptor)	19	μM^-1h-1^
k_off_ (first order rate constant for dissociation of drug-receptor complex)	0.15	1/h
Baseline	0	
slope	–33	mmHg

### CASE STUDY 3: *IN VITRO IN VIVO* EXTRAPOLATION OF DIFFERENCES IN PD

The third case study investigates the application of PBPK linked to a semi-mechanistic PD model to predict the response to a drug based on clinical data for a different drug that acts on the same target. Such models are useful for comparing several compounds that belong to the same therapeutic class. Semi-mechanistic PD models combine mechanistic aspects of the PD relationship with empirical features and are commonly used where the mechanism of drug action is not fully understood or when there is insufficient data available to develop a fully mechanistic model. The operational model of agonism (equation 1) was developed from receptor theory to describe *in vitro* pharmacology (Black and Leff, 1983) and has also previously been applied in PKPD modeling (Van der Graaf et al., 1997, 1999; Cox et al., 1998; Cleton et al., 1999, 2000; Zuideveld et al., 2004; Jonker et al., 2005).

(1) $$E=\frac{E_{m}\cdot \tau^{n}\cdot {\left[A\right]}^{n}}{{\left(K_{A}+\left[A\right]\right)}^{n}+\tau^{n}\cdot {\left[A\right]}^{n}}\,\;\;\;\;\;\;\;\;\;\;\;\;\;\;\;\;\;\;\;$$

Mechanistic features are incorporated in terms of drug binding affinity (K_A-_ which represents the binding affinity of drug A to the receptor) and intrinsic efficacy (ε proportional to the transducer ratio τ;), both drug-dependent parameters for which information can be measured *in vitro*. The conversion of receptor activation to the PD response is described empirically by the system-dependent parameters E_m_, the maximum effect achievable in the system, n, the slope of the occupancy effect relationship and τ, which is related to the receptor concentration transduction properties of the tissue. System-dependent parameters are shared for drugs with the same mechanism of action in the same system. The operational model of agonism has previously been used to describe the PD effect of benzodiazepines in animal models (Cleton et al., 1999, 2000).

In this example, the operational model of agonism was used to describe the hypnotic effects of triazolam, as measured by change in beta-EEG amplitude, and to extrapolate the model to zolpidem by changing only the drug related parameters of the PBPK-PD model. The hypnotic effects of both zolpidem and triazolam are mediated via the same binding site on α1 subunit containing GABA_A_ receptors. Triazolam and zolpidem were selected for two reasons. Firstly, the PD effect is related to the concentration of the parent compound only; zolpidem has no active metabolites, while, the active metabolite of triazolam is rapidly metabolized and thus does not contribute significantly to activity. Garzone and Kroboth (1989) Secondly, several clinical PK and PD studies have been reported for both compounds by the same group, which is important for the PD response since there is no standardization of the measurement and analysis of EEG recordings used as the PD effect measure, making it difficult to pool and compare data collected by different research groups. In this example, clinical data is used to establish the model for triazolam only, thus zolpidem is treated as a compound in pre-clinical development, with published clinical data used only to confirm the accuracy of the modeling approach.

Simulations of triazolam and zolpidem PK and PD were performed in virtual Caucasian healthy volunteers (HVs) and the Simcyp Triazolam (**Table 3**) and Zolpidem (**Table 4**) compound files with the first order absorption model, minimal PBPK distribution model and clearance described by enzyme kinetics. Unbound plasma concentration was used as the input to the PD model.

**Table 3:** Parameters used for the triazolam PBPK model (Simcyp V12).

Parameter	Value	Unit
Molecular weight	343.2	g/mol
LogP	2.42	
Compound type	Ampholyte	
pKa 1	10.52	
pKa 2	2.91	
Blood:plasma	0.625	
Fu plasma	0.179	
Main binding protein	albumin	
Absorption	First order absorption	
fa	1	
ka	1.175	1/h
Distribution	Minimal PBPK model	
Vss	0.54	L/kg
**Elimination**		
CYP3A4:1-OH metabolite: Km	15.6	μM
CYP3A4: 1-OH metabolite: Vmax	4.35	pmol/min/mg microsomal protein
CYP3A5: 1-OH metabolite: Km	23.8	μM
CYP3A5: 1-OH metabolite: Vmax	8.18	pmol/min/mg microsomal protein
CYP3A4:4-OH metabolite: Km	176.0	μM
CYP3A4: 4-OH metabolite: Vmax	11.5	pmol/min/mg microsomal protein
CYP3A5: 4-OH metabolite: Km	142.0	μM
CYP3A5: 4-OH metabolite: Vmax	12.5	pmol/min/mg microsomal protein
CL_R_ (renal clearance)	0.274	L/h

**PD model**	Operational transduction model	

Unit 1:	
Emax	1.00
Dissociation constant	0.001
Baseline	0
Unit 2:	
Maximum effect achievable in the system (E*m*)	2.08
Slope of the occupancy effect relationship (n)	1.81
Transducer ratio (τ)	1.76

**Table 4:** Parameters used for the zolpidem PBPK model (Simcyp V12).

Parameter	Value	Unit
Molecular weight	307.39	g/mol
LogP	2.42	
Compound type	Monoprotic base	
pKa 1	6.16	
Blood:plasma	0.76	
Fu plasma	0.08	
Main binding protein	albumin	
Absorption	First order absorption	
fa	1	
ka	2.25	1/h
Distribution	Minimal PBPK model	
Vss	0.68	L/kg
**Elimination**		
CYP3A4:Metabolite 4: Km	340	μM
CYP3A4: Metabolite 4: Vmax	1.41	pmol/min/mg microsomal protein
CYP3A4: Metabolite 11: Km	399	μM
CYP3A4: Metabolite 11: Vmax	6.86	pmol/min/mg microsomal protein
CYP1A2:Metabolite 4: Km	40	μM
CYP1A2: Metabolite 4: Vmax	0.777	pmol/min/mg microsomal protein
CYP2D6:Metabolite 4: Km	214	μM
CYP2D6: Metabolite 4: Vmax	4.68	pmol/min/mg microsomal protein
CYP2C9:Metabolite 4: Km	81	μM
CYP2C9: Metabolite 4: Vmax	0.888	pmol/min/mg microsomal protein
CL_R_ (renal clearance)	0.18	L/h

**PD model**	Operational transduction model	

Unit 1:	
Emax	1.00
Dissociation constant	0.053
Baseline	0
Unit 2:	
Maximum effect achievable in the system (E*m)*	2.08
slope of the occupancy effect relationship(n)	1.81
Transducer ratio (τ)	1.76

Equilibrium dissociation constant (K_A_) values for triazolam (1 nM) and zolpidem (53 nM) and relative intrinsic efficacy of the two compounds (Equation 1) were identified from published *in vitro* data (Garzone and Kroboth, 1989; Hadingham et al., 1993; Van der Graaf et al., 1999; Zuideveld et al., 2004). Since triazolam and zolpidem have comparable efficacy, the transduction ratio (τ) was assumed to be the same for both compounds. Simcyp Parameter Estimation module (using weighted least square objective function and Nelder–Mead optimization methods) was used to determine the values of τ, Em and n in addition to the effect compartment elimination rate (keo) to account for hysteresis in the response, using published data (Smith et al., 2001; Sancar et al., 2007). The weighted mean of the estimated values of each parameter was used in the simulations.

The quality of these parameter estimates to predict the PD response to triazolam was tested by the ability of the model to predict the PD response following interaction with the CYP3A inhibitor ketoconazole (von Moltke et al., 1996; Greenblatt et al., 1998). Thereafter, the dose of zolpidem predicted to produce the equivalent response to 0.25 mg oral triazolam (based on the maximal response and the area under the effect curve between 0 and 12 h) using the PBPK-PD model developed for triazolam and the K_A_ and intrinsic efficacy for zolpidem, was identified using the Simcyp automated sensitivity analysis module for the dose range 0.01–1000 mg oral zolpidem. This was then compared with the clinically applicable dose for verification of the PBPK/PD model.

### CASE STUDY 4: UNDERSTANDING THE COVARIATES DETERMINING PD VARIABILITY

The fourth case study explores the ethnic differences in the QTc prolongation by quinidine using the target tissue (heart) drug concentrations. It has been suggested that in specific situations, PBPK/PD models are more likely to allow a better understanding of true PD variability versus variability resulting from drug disposition alone (Rostami-Hodjegan, 2013), which is usually reflected by plasma concentrations. Quinidine is known to cause lengthening of the QT interval in the electrocardiogram (ECG), with greater potential for QT prolongation in females (Benton et al., 2000; El-Eraky and Thomas, 2003; Shin et al., 2007). Ethnic differences in QT prolongation have also been demonstrated (Shin et al., 2007), with greater QT prolongation observed in Caucasian females than in Korean females, despite no significant differences in plasma concentrations. These differences in QT prolongation may be of significance clinically since lengthening of the QT interval corrected for heart rate (QTc) that is >500 ms is believed to be a contributory factor to the life-threatening side effect of Torsades de pointes observed with some drugs (Bednar et al., 2001).

Traditional PK/PD models linking plasma concentrations to QT changes in Caucasians and Koreans have reported a higher Emax (the maximum value of QTc changes) values in Caucasian females with similar EC_50_ (concentration of quinidine required to produce 50% of the maximum response) values in both ethnic groups, suggesting similar sensitivity to quinidine concentrations in the two groups (Shin et al., 2007). PBPK/PD modeling using free heart concentrations of quinidine that may be more relevant to the QT prolongation effect of the drug may have a greater potential to provide an understanding of the ethnic differences in the observed QTc changes.

Data from the study by Shin et al. (2007) were used to develop the PBPK/PD model, with virtual Caucasian HV and virtual Chinese HV (to represent Korean). The Simcyp compound file for quinidine (**Table 5**), a full PBPK distribution model with first order absorption and clearance of quinidine of 19.4 (CV 38%) L/h in Caucasians and 18.16L/h (34%) in Koreans was used. This PBPK model was verified by comparison of the plasma concentration versus time profile with clinical data. The Emax model used the measured mean baseline QTc of 443 ms for Koreans and 445 ms for Caucasians (Shin et al., 2007). Input to the PD model was predicted free heart concentrations and parameter estimation was used to estimate ΔEmax and EC_50_. EC_50_ was used as a marker of sensitivity and compared in the two groups of virtual subjects.

**Table 5:** Parameters used for the Quinidine PBPK model (Simcyp V12).

Parameter	Value	Unit
Molecular weight	324.4	g/mol
LogP	2.88	
Compound type	Diprotic base	
pKa 1	4.2	
pKa 2	8.8	
Blood:plasma	0.88	
Fu plasma	0.203	
Main binding protein	albumin	
Absorption	First order absorption	
fa	1	
ka	3	1/h
Distribution	Full PBPK model	
Vss	1.16	L/kg
**Elimination**		
CLiv	19.4 Caucasians 18.16 Chinese (Korean)	L/h
CL_R_	1.95	L/h

**PD model**	Simple Emax model	

Emax	Parameter estimation used for fitting to clinical data	ms
EC50	Parameter estimation used for fitting to clinical data	μM
Baseline	443 Korean 445 Caucasian	ms

## RESULTS

### CASE STUDY 1

**FIGURE 1:**
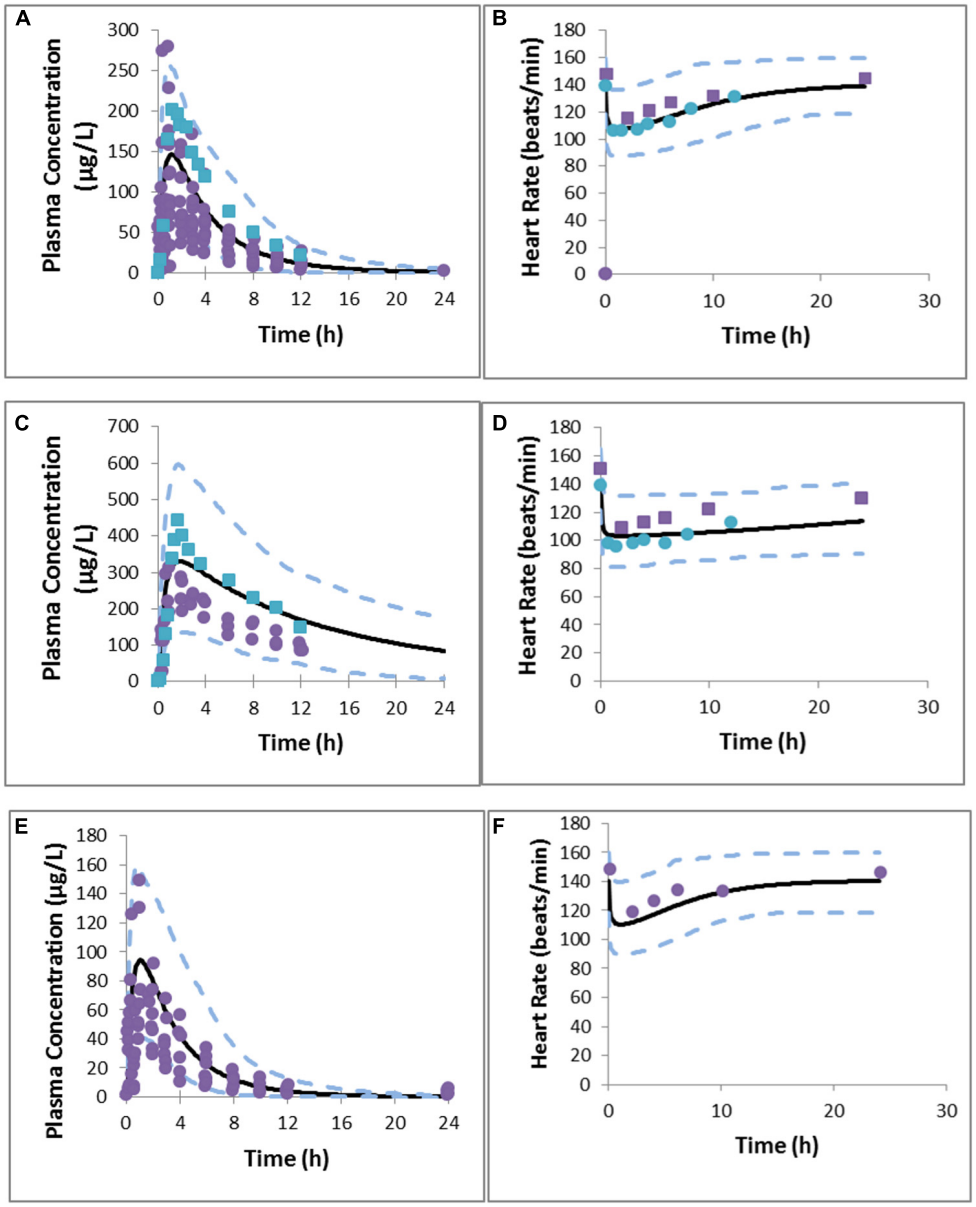
**Predicted and observed metoprolol plasma concentration profile in EMs **(A)**, PMs **(C)**, UMs **(E)**, PD response in EMs **(B)**, PMs **(D)**, and UMs **(F)**.** Simulations are presented as the mean of 10 trials (bold black line) and 95% confidence interval (dashed line). Solid circles indicate observed values reported by Kirchheiner et al. (2004) and squares represent values reported by Sharma et al. (2005).

In general both PK and PD profiles were predicted successfully, as is evident from **Table 6** that summarizes the PK and PD parameters and **Figure 1** where the simulated data has been superimposed on observed data (Shimada et al., 1996; Kirchheiner et al., 2004). These models successfully simulated PK and PD profiles of metoprolol and support the potential for prediction of genetic differences in PD once the PKPD relationship is established in wild-type genotypes.

**Table 6:** Observed vs. predicted “PRED” Metoprolol PK/PD parameters in healthy volunteers by CYP 2D6 metabolizer status.

	PM			EM			UM		
	PRED	Observed	Ratio	PRED	Observed	Ratio	PRED	Observed	Ratio
**PK parameters**									
AUC (ug/L/h)	4,938	3,921	1.26	586	839	0.70	304	273	1.1
Tmax (h)	1.82	1.63	1.12	1.18	1.35	0.88	1	1	1.1
Cmax (ug/L)	305	363	0.84	112	178	0.63	69	67	1.0
CL/F (L/h)	20	24	0.85	171	139	1.22	329	367	0.9
**PD parameters**									
Rmax (beat/min)	142	151	0.9	142	149	1.0	142	148	1.0
Rmin (beat/min)	103	109	0.9	109	116	0.9	113	119	0.9
t(Rmin) (h)	1.9	2	1.0	1.2	2	0.6	1.2	2	0.6
AUC (beat.h/min)	831	685	1.2	328	363	0.9	223	308	0.7


The simulated CL (Dose/AUC) of the UM group was found to be 16- and 2-fold higher than that of PM and EM groups, respectively, suggesting that UMs may not achieve adequate therapeutic response on a standard dose of 100 mg metoprolol. Simulated mean PD profiles showed that the area under the effect curve in PMs was 4-fold higher than that in UMs, and 2-fold higher than that in EMs. The simulated/observed ratios for the maximum reduction in heart rate and absolute area under effect curve are 0.94 and 1.2 for PMs, 1.0 and 0.94 for EMs, and 0.96 and 0.73 for UMs groups, respectively.

It is clear from these results that the status of CYP2D6 phenotype has an impact on the reduction in heart rate. PMs are of particular interest as the PD effect is higher and takes longer to return to the initial point. In comparison with EMs, and UMs, the longer action of metoprolol in PMs is a result of residence of drug in the body (see plasma concentration profile for PMs), which is caused by the lower clearance of metoprolol in PMs group. These differences indicate significant effects on metoprolol dosing in the corresponding groups of patients which could have been predicted *a priori.*

Simulation results showed consistency with clinical observations in terms of significant differences of metoprolol PK/PD profiles between PMs and UMs with a marginal change between EMs and UMs. UMs may not achieve optimal target concentrations of metoprolol, which can lead to a lower benefit from the standard 100 mg dose of the drug compared with PMs.

### CASE STUDY 2

Predicted PK and PD profiles for IR nifedipine in Japanese hypertensive subjects suggested that the model was successful in recovering the clinical data (Kikuchi et al., 1982). Comparison of the PK and PD parameters (Cmax and Rmax respectively) showed that the predicted Cmax/observed Cmax and predicted Rmax/observed Rmax are within the 2-fold acceptability criteria (**Table 7**).

**Table 7:** Comparison of the predicted and observed Cmax and maximum reduction in systolic blood pressure (Rmax) for the different nifedipine formulations and doses.

	10 mg IR nifedipine			60 mg nifedipine GITs			30 mg nifedipine GITS first dose			30 mg nifedipine GITS final dose		
	Pred	Obs^a^	Ratio Pred/Obs	Pred	Obs^b^	Ratio Pred/Obs	Pred	Obs^c^	Ratio Pred/Obs	Pred	Obs^c^	Ratio Pred/Obs
C_max_ (ng/ml)	127.8 ± 53.6	132.5 ± 23.7	0.96	44.3 ± 22.6	31.0	1.42	38.1 ± 31.5	16.9 ± 10.2	2.25	56.6 ± 51.1	30.7	1.84
R_max_ (mmHg)	–30.9 ± 3.6	–32.9 ± 9.9	0.94	–25.1 ± 5.5	–23.0	1.09	–24.3 ± 7.8	–13.7 ± 15.6	1.77	–26.0 ± 5.1	–19	1.37

*Data are reported as the mean ± SD (where reported). Observed values are from (a) Shimada et al. (1996), (b) Meredith and Elliott (2004), and (c) Brown and Toal (2008).*

**FIGURE 2:**
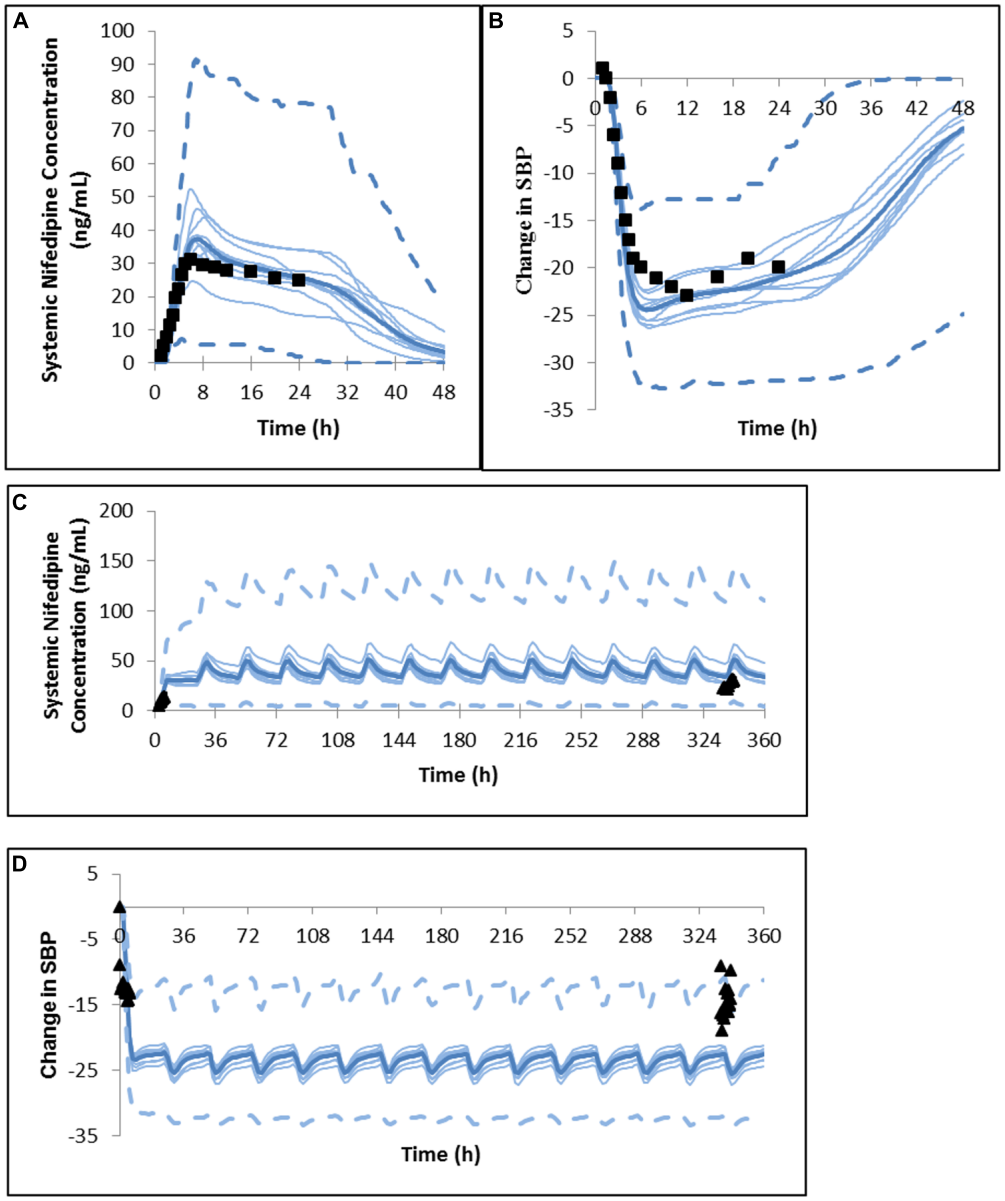
**Predicted and observed **(A)** plasma concentration profile and **(B)** change in systolic blood pressure after a single dose of nifedipine 60 mg GITS in North European hypertensive subjects (Meredith and Elliott, 2004).** Predicted and observed **(C)** plasma concentration profile and **(D)** change in systolic blood pressure after the initial dose and daily dosing of nifedipine 30 mg GITS for 15 days in North European hypertensive subjects (Brown and Toal, 2008).

Both the magnitude and sustained plateau (>24 h) of the PK and PD profiles were well captured for 60 mg nifedipine GITS formulation, with mean clinical data falling within the range of the mean values of simulated trials (**Figure 2**). The comparative PK and PD ratios in **Table 7** also confirm the successful prediction of the PK/PD profile of the 60 mg GITS formulation, for which a rich *in vitro* data set was available.

However, for a 30 mg multi-dose study of nifedipine GITS, visual inspection suggests that PK and PD is overpredicted (**Figure 2**), although the majority of observed values are within the 5 and 95% percentiles of the predicted profiles. Based on comparative ratios (**Table 7**) Cmax was marginally overestimated after the first dose, with a Cmax (predicted)/Cmax (observed) ratio of 2.25 and the other parameters within 2-fold of the observations.

It is notable that in this study no parameter fitting based on clinical data was used, with the aim of mimicking a situation in which prediction of the formulation effect is based on the use of *in vitro* data for propagation and the prediction of the PK and PD profiles.

### CASE STUDY 3

**FIGURE 3:**
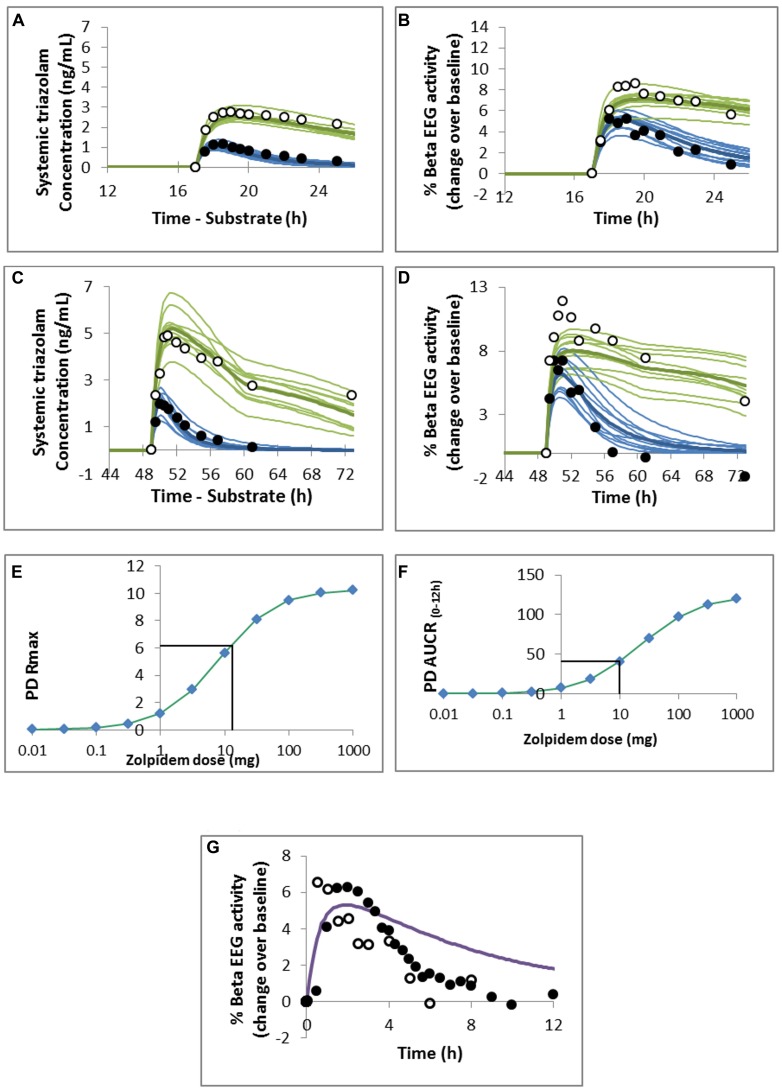
**Predicted and observed **(A,C)** triazolam plasma concentration profile and **(B,D)** pharmacodynamic response to **(A,B)** 0.125 mg or **(C,D)** 0.25 mg oral triazolam in the absence (closed circles and blue lines) or presence (open circles and green lines) of ketoconazole.** Observed data are from **(A,B)** von Moltke et al. (1996) and **(C,D)** Greenblatt et al. (2000) and the simulated study designs were matched to these studies. Predicted maximal observed response (PD Rmax; **E**) and area under the response curve (AUCR_0-12_; **F**) for a range of doses of oral zolpidem. The mean value of the response measure for 0.25 mg oral triazolam is indicated by the horizontal black line and was used to estimate the dose of zolpidem resulting in the equivalent measure of PD response. **(G)** Predicted mean PD response to 10 mg oral zolpidem. Observed data points are from Greenblatt et al. (2000; closed circles) and Greenblatt et al. (2006; open circles).

Fitted values of E_m_, τ, n, and k_eo_ were 20.6, 1.0, 0.93, and 2.0 respectively. The resulting model was able to predict the PD response to 0.125 and 0.25 mg triazolam with and without ketoconazole DDI reasonably well as seen in **Figure 3**, although the PD response was underestimated at the highest plasma concentrations of triazolam (**Figure 3D**).

A dose of ∼10–13 mg zolpidem was predicted to result in the same maximal response (R_max_) and area under the effect curve as a 0.25 mg dose of triazolam (**Figures 3E,F**). Visual inspection of the concentration – effect curve shows a good prediction of the maximum effect of zolpidem but the duration of the effect is overestimated (**Figure 3G**) compared with the clinical data (Greenblatt et al., 2000, 2006).

### CASE STUDY 4

**FIGURE 4:**
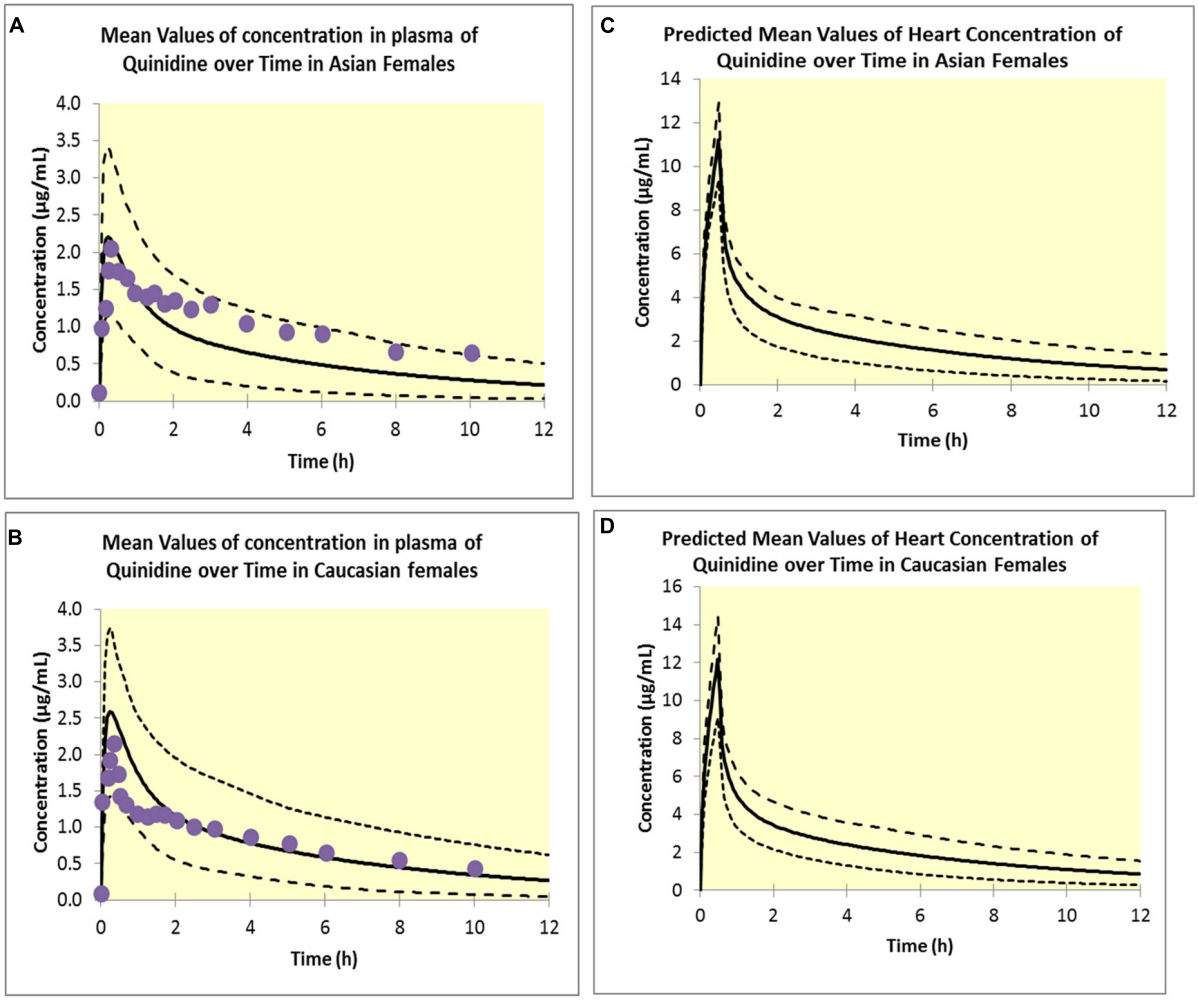
**Simulated plasma concentrations in Asian females **(A)** and Caucasian females **(B)**.** Solid lines represent mean values; dotted lines represent the upper and lower confidence intervals and solid circles represent observed data (Shin et al., 2007). Predicted free heart concentrations in Asian females **(C)** and Caucasian females **(D)**. Solid lines represent mean values; dotted lines represent the upper and lower confidence intervals.

The PBPK model predicted clinically observed plasma PK profiles of quinidine in Caucasian and Korean (represented by Chinese HVs) females (Shin et al., 2007) adequately as verified by visual predictive checks (**Figures 4A,B**)). Simulations of free heart concentrations of quinidine over time for both groups are shown in **Figures 4C,D.**

**FIGURE 5:**
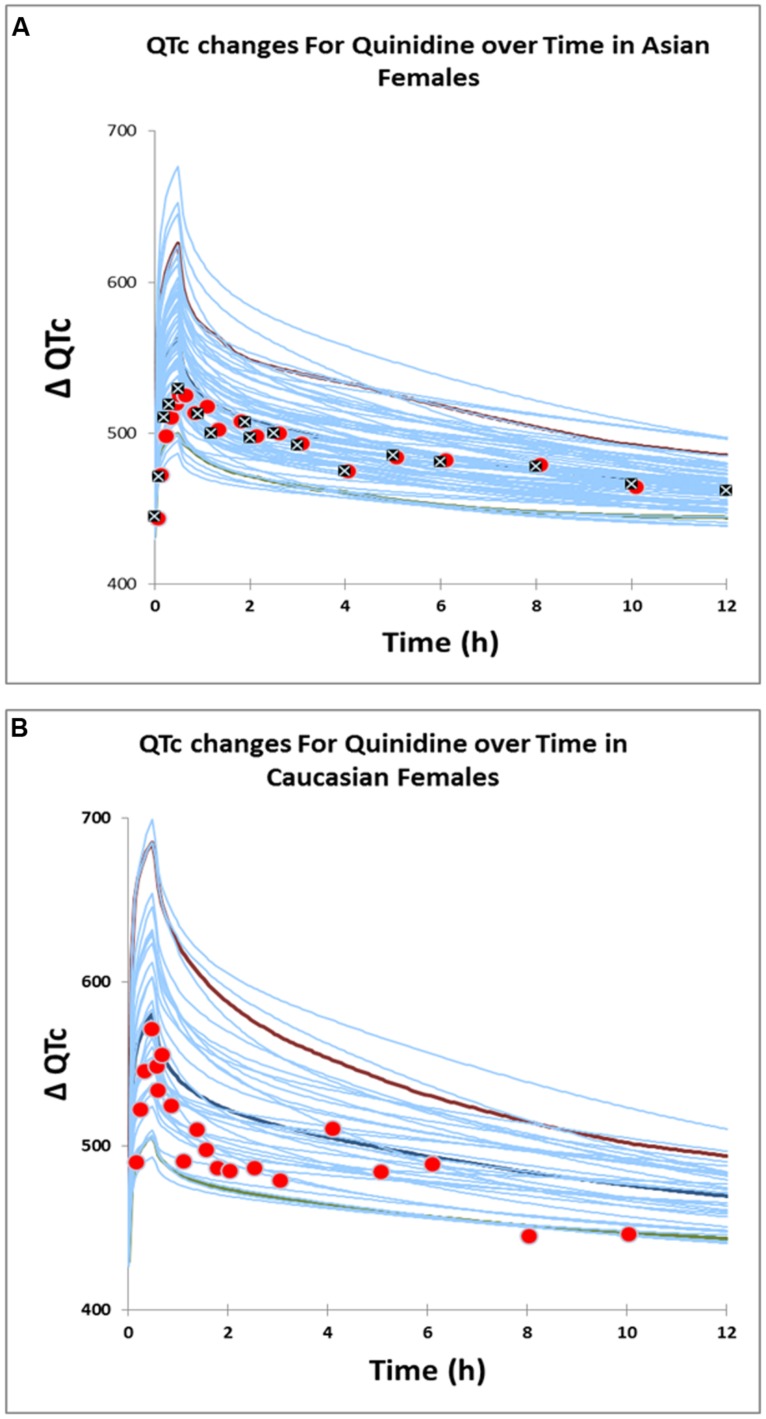
**Predicted QTc changes in Asian females **(A)** and Caucasian females **(B)**.** Solid lines represent profiles in virtual individuals. Solid circles represent observed data (Shin et al., 2007) in Caucasians and Asians. Solid squares represent observed data (Kim et al., 2007) in Asians.

Estimated E_max_ and EC_50_ values were 190.0 ms and 1.53 μM respectively in Caucasian females and 175.19 ms and 1.80 μM respectively in Korean females. Visual predictive checks of the simulations suggested that these PD models recovered the greater QTc prolongation observed clinically (Kim et al., 2007; Shin et al., 2007) in Caucasian females adequately (**Figure 5**).

The estimated sensitivity parameters (EC_50_) showed a Caucasian:Korean ratio of 0.85, indicating a greater sensitivity to heart quinidine concentrations in Caucasian females. This suggests that a standard dose of quinidine has the potential to produce QTc in more Caucasian females than in Korean females because of this difference in sensitivity.

## DISCUSSION

Recent advances in IVIVE coupled PBPK models have facilitated informed covariate recognition of the observed PK variability. Further, these models allow connecting response to the unbound drug concentrations at the site of action which in turn improves our ability to link the concentration-response relationships beyond merely relying on the plasma concentration as the response’s driving force. Connecting PD models, even empirical or semi-mechanistic ones, to PBPK models is the natural progression after developing predictive PK models.

To illustrate the added value in utilizing PBPK/PD models four case studies are presented here. These have demonstrated that linking a PD model to a PBPK model allows the prediction of the effects of a change in metabolizing enzyme phenotype, drug formulation, drug receptor binding, or ethnic differences in sensitivity to the drug on the PD response through propagation of the change in PK. Such models may assume that the concentration-response relationship remains unchanged when the PK changes occur. It is recognized that this is not always the case, and any mismatch between predictions and observations may provide additional information about the mechanism of action of a drug and covariates relevant to the PD responses. Such factors can then be investigated and built into the models.

The first case study illustrates the potential for prediction of genetic differences in PD once the PKPD relationship is established in wild-type genotypes. Although population pharmacokinetic (POPPK) studies have been valuable in informing investigators of PKPD differences associated with different phenotypes, these studies need to be powered adequately to recognize such differences. Clinical trial simulations similar to the one shown in this case study can also be used to investigate the design of studies and their power to ensure that less frequently occurring phenotypes that are predicted to be relevant to dose evaluation are included. It is noteworthy that such simulations can be used in lieu of clinical studies to inform drug labels. (Janssen Therapeutics, 2013).

The second case study demonstrates that integration of a PBPK model that accounts for differences in formulation effects with a dynamic PKPD binding model predicts differences in clinical PD observations reasonably well, a feature that is very challenging to implement in classical compartmental approach. Prediction of the formulation effect based on *in vitro* data is propagated to the prediction of the PK and PD profiles, without the use of parameter fitting to observed data. The marginal over-prediction of the plasma profile can, in a large part, explain the over-prediction of the PD response that was observed in the study by Brown and Toal (2008). Overestimation of the plasma profile and PD response to 30 mg nifedipine GITS in this study may relate to differences in the dissolution profile of the batch of 30 mg GITS tablets used in the clinical study (which was not reported), compared to the dissolution profile used for simulation. In the absence of a published dissolution profile for the 30 mg GITS tablets, a dissolution profile proportional to the 60 mg GITS tablets were assumed for the simulations. It might have been expected that a PD model for the hypotensive response to nifedipine developed for and IR would underestimate the response to CR of nifedipine. Rate of increase in the plasma nifedipine concentration has been shown to influence the haemodynamic response, with a more rapid increase associated with increased sympathetic nervous system activation, increased heart rate and a diminished reduction in blood pressure response (Kleinbloesem et al., 1987; Meredith and Elliott, 2004; Brown and Toal, 2008). This may not have been the case since no increase in heart rate in hypertensive subjects was observed in the study from which Shimada et al. (1996) took the nifedipine PK and PD data to develop the PKPD model that was used in this case study (Kikuchi et al., 1982).

In the third example, by changing only the PBPK model input and the K_A_ value for zolpidem, a good estimate of the dose requirement of zolpidem was obtained in HVs. The dose estimated using this PBPK/PD model is in agreement with the recommended dose of 10 mg zolpidem in adults. However, when compared to clinical data (Greenblatt et al., 2000, 2006), although the maximal response to zolpidem was predicted reasonably well, the duration of the response was overestimated. A possible explanation could be the clockwise hysteresis observed in the clinical data for zolpidem, suggesting acute tolerance effects to zolpidem, as has previously been proposed (de Haas et al., 2010). However, clockwise hysteresis is not observed for triazolam, suggesting that differences in the mechanism of action between zolpidem and triazolam may not be fully accommodated by the model. This example demonstrates the application of a combined PBPK and semi-mechanistic PD model in predicting the response to a compound based on clinical data for a different compound that acts at the same target.

Results in the fourth example demonstrate that the PBPK/PD model that used unbound heart concentrations of quinidine to drive the changes in QTc prolongation was effective in recovering the clinically observed ethnic difference in QTc prolongation. This model, which was of greater physiological relevance than previously published models, enabled us to gain a plausible mechanistic explanation for the observed ethnic differences in QTc prolongation, despite the similarities in the measured plasma concentrations in the two population groups. The higher EC_50_ in Asian females illustrate that this group is less sensitive to the QTc prolongation effects of quinidine and require higher free concentrations of quinidine at the target site to produce an equivalent change in QTc prolongation. Further studies to elucidate the mechanistic basis for the differences in sensitivities and also to investigate the potential contribution of 3-hydroxy quinidine (a primary metabolite that may contribute to pharmacological activity) are warranted.

Knowledge of inter-patient variability in response to drugs is crucial during drug development and clinical practice. PBPK/PD models such as the ones presented above provide a seamless framework to assess the propagation of key PK variables resulting from differences in physiology, genetics, demographics, concurrent medications, different formulations, etc. through to PD effects. Predicting such variability using the ‘bottom up’ approach prior to planning clinical trials enables researchers to optimize study design and predict results that are likely to be more reflective of the general population using the drug. Furthermore, when measured plasma concentrations cannot be reliably correlated with PD effects, PBPK/PD offer a valuable alternative to traditional compartmental modeling.

## AUTHOR CONTRIBUTIONS

Manoranjenni Chetty, Rachel H. Rose, Khaled Abduljalil, Theresa Cain, Gaohua Lu, and Nikunjkumar Patel designed and performed the research and analyzed the data. Manoranjenni Chetty, Rachel H. Rose, Khaled Abduljalil, Masoud Jamei, and Amin Rostami-Hodjegan wrote the manuscript.

## Conflict of Interest Statement

Manoranjenni Chetty, Rachel H. Rose, Gaohua Lu, Theresa Cain, Khaled Abduljalil, Nikunjkumar Patel and Masoud Jamei are employees of Simcyp Limited (a Certara company). Amin Rostami-Hodjegan is an employee of the University of Manchester and part-time secondee to Simcyp Limited (a Certara Company).
